# American Academy of Dental Science

**Published:** 1899-06

**Authors:** 

**Affiliations:** American Academy of Dental Science


### Reports of Society Meetings.

### AMERICAN ACADEMY OF DENTAL SCIENCE.

The regular monthly meeting of the American Academy of Dental Science was held at Young's Hotel, Wednesday evening, January 4, 1899, at six o'clock.

A paper was read by Frank A. Delabarre, D.D.S., M.D., of Boston, entitled "The Power of Adaptation to Environment as shown by Cells and Tissues in Various Pathological Processes."

(For Dr. Delabarre's paper, see page 359.)

DISCUSSION.

*Dr. Eames.*—The ideas expressed in regard to the wonderfulness of pathological action are in line with the thoughts which came to me in my study of pathology. The essayist referred to the chemotactic action of the blood-cells, the white corpuscles having the power of moving to any point to attack a foreign substance in the blood, such as pyogenic bacteria and poisonous substances, and, what has not been mentioned, the fact that when a substance, such as pulverized glass, which has no pyogenic action, is inserted into the circulation, these white blood-cells are not attracted to it and yet they are attracted to other foreign substances which are injurious. It is that power to discriminate between the harmless and the harmful substance which strikes me as being a very wonderful thing.

*Dr. Williams.*—I was pleased with the paper, which appeared to me to be very concise and to very well represent the pathological side of medicine and surgery. The essayist's statement of the impression which the study of pathology had made upon him reminded me of the impression it made on a very distinguished old physician of Boston, who is now dead, who, after long study, came to the conclusion that disease was an institution of nature; that it was foreordained, as a part of the plan of creation. I suggested to him that there was another way of looking at it, which I believed to be the correct way, and that is, that in the plan of creation the provisions made for the continuance of life were so strongly fixed in the way of health as to give laws to, and control disease, and to attempt to repair injury. I think if we study the action of the human system under pathological conditions and in cases of wounds and fractures, we will conclude the plan of creation was along the line of health, and is so firmly fixed along that line as to resist disease or to repair injury to a degree which in some cases is marvellous.

*Dr. Brackett.*—I came in too late to hear all of the paper. That portion which I did hear I very greatly admired, as the idea which was made prominent in the paper is an idea that has impressed me very much for many years. It is an old saying, quoted I think from Young, that "the undevout astronomer is mad." This, being expressed in other words, means, if my interpretation is correct, that any man who considers the stupendous scheme of the universe, who studies the motions of the heavenly bodies, the grandeur and awful immensity of space, who investigates all these laws of time, motion, and position without being impressed with the nobility of the whole scheme, must be very dull and dwarfed in intellect. And since so much of the study of recent years of medical and scientific men has been in the world of small things, and with the microscope, it has seemed to me that the man who has given any attention to anything of this field of human investigation without being impressed with the beauty and the nobility of the plan of creation must be very unimpressionable. Just as truly as astronomers have for generations been impressed with the grandeur and immensity of the plan of creation as shown in the existence, position, and motion of the innumerable heavenly bodies, just so truly has the thoughtful man of recent years been impressed with the wonderfulness and importance of minute things in all that goes to make up a world in its broadest sense. There is in all of that material that which should inspire our liveliest appreciation and our worthiest feelings; and it should be an inspiration for all of us as human individuals, occupying legitimately a place on this earth, and having a part assigned to us, to do our part with as much faithfulness and promptness and decisiveness as does the white corpuscle, or as does the principle of gravitation, or as does the shining of the sun, or the law of combustion or chemical action, or any of the physical laws which act with unerring certainty. The consideration of all these things should be an example and inspiration to us in the performance of those duties which are so plainly before us.

*Dr. Delabarre.*—The chemotactic action which is exhibited by the different cells of the body is similar to a chemical action, as it is both positive and negative; that is to say, the white blood-corpuscles have the peculiarity of being attracted by certain substances, and they are repelled by other substances. They are attracted by bacteria, alkaloids, and albuminates, and make a very strong effort to overcome their effects.

### DISCUSSION ON PORCELAIN INLAYS.

*Dr. Hamilton.*—While I have always been interested in porcelain inlays since seeing some remarkably good ones done by Dr. Ames, of Providence, nearly twenty years ago, yet I have done but few of them, and am here to-night to show the Jenkins apparatus which Dr. Abbot, of Berlin, presented to Dr. Hadley and myself, rather than as an expert in the work.

From the dentist's stand-point inlay work has not before been brought to the degree of perfection that most of our other operations have been, and so has been unsatisfactory, but it has, as a rule, pleased the patients. It is remarkable how well a poor inlay looks at a distance of a few feet.

The Jenkins system consists of a new body, low fusing but of great strength and sharp edges, and a great variety of good colors. Then there is a very neat furnace, all beautifully gotten up so that the inlays can be made in the operating-room.

The impressions of the cavity are taken in No. 30 or No. 40 gold-foil and embedded in a paste of powdered asbestos. After drying this investment, the body, mixed with absolute alcohol, is put in the matrix and fired. For small inlays more body is added and fired a second time, and for a large one a third firing is necessary. This should bring the inlay to the proper contour so that no surface grinding is necessary, although the body takes a good polish.

*Dr. Williams.*—I would like to inquire whether this body is fused at a higher or lower temperature than the Downie?

*Dr. Hamilton.*—The Downie is quite a degree higher. You can use the Jenkins body in a Downie furnace.

*Dr. Werner.*—How much shrinkage is there in the average case in the way you have up to the present learned to manipulate it?

*Dr. Hamilton.*—The shrinkage of the first firing is considerable; then the body added for the second firing fills the crevice at the edge. The third firing gives contour.

*Dr. Werner.*—How do you add the extra body?

*Dr. Hamilton.*—Put on the body in paste form with a small brush or a spatula. You do not disturb the inlay after the first firing. It does not pull away from the matrix, so that your fit is very exact.

*Dr. Wilson.*—I have lately seen a patient just from Dr. Jenkins's office, and have had the opportunity of seeing some very handsome inlay work. It must take a great deal of time and patience to bring about the results obtained by Dr. Jenkins, and it would seem to me, in many cases where an exact impression of the tooth could be taken, much time would be saved, more especially in cavities running beyond the margin of the gum. Dr. Jenkins inserts a good many inlays in this class of cavities very skilfully, and I am curious to see how long they will last. I think the statement has been made that this body is harder than the teeth furnished by the Dental Depots. This I do not think is possible, although I have no doubt it is sufficiently strong for practical purposes in doing ordinary inlay work.

*Dr. Briggs.*—I have always been very much interested in porcelain fillings, and some time ago I had occasion to do some work for a patient who, like the one referred to by Dr. Wilson, had been in Dr. Jenkins's hands. I was very much struck not only with the beauty and match in color of these fillings, but with the fit in inaccessible places, which I realized I had very much difficulty in trying to get with such methods and materials as came within my knowledge. I wrote to Dr. Jenkins, complimenting him on the result he had obtained and asking him how he did it. In reply I got a letter outlining this process of his, and also stating at that time that he was very sorry that he could not supply any to anybody in this country. About the same time I received a circular from the manufacturers describing this process. Through the kindness of Dr. Hamilton, who let me have some of the enamel, I have been enabled to do a little experimenting in connection with this subject, and my summing up of his outfit and process, as it appears to me, would be something like this: That he has given us the ability, by using this thin gold, to make a matrix of the cavity which is practically perfect; and he has given us an enamel that has excellent color and that flows in this matrix without destroying it, and by repeated fusings can be made to fit the matrix perfectly. If one makes these other enamels or bodies in a platinum matrix, they adhere to the platinum, and many times your filling is spoiled, but with the Jenkins enamel this gold matrix is readily removed and you have a perfect filling. This may be due partly to the technique, the mechanical part, but a great deal of it comes through the make-up of his enamel. You cannot do it with a high-fusing body, because the gold melts before the body is biscuited. You cannot do it with the low-fusing bodies that we have in the market, because when they are done they do not look like anything that was ever in the human mouth. I deplore the fact that I cannot get hold of the Jenkins enamel, even if I could not get the apparatus, for I feel that if I had that enamel I could secure very good results by using some furnace which was not quite so dainty as Dr. Jenkins's. I wish he would publish of what the enamel is composed.

In regard to taking impressions for porcelain fillings referred to by Dr. Wilson, I have done a great many in that way, and have had the fillings baked by others,—Dr. Moffatt has made some for me. Still you never get an impression of a cavity that is perfect. When the inlays come to you they have to be touched up and ground, and I have never been able to get as perfect a fit as I would like. As far as the strength of these enamels is concerned, they seem to me exceedingly strong. I think Dr. Jenkins has found something that is very useful to us.

*Dr. Daly.*—I have read about Dr. Jenkins's method of making and inserting porcelain fillings with a great deal of interest. I regretted that the outfit was not to be obtained at once, and I congratulate those gentlemen who have been fortunate enough to secure one.

The method which I employ is one that I do not think will be of any interest to you, as it is very crude, for I have been disappointed in the Downie furnace with the rest of you, because after burnishing my platinum-foil for the matrix, and placing in that the body in anticipation of getting a filling that will be perfect in color, when it is fired many times you cannot help feeling that it is "all a delusion and a snare," for instead of your perfect color you oftentimes get a translucent bit of porcelain more like little drops of glass. After getting this result and cracking it a dozen times or so, I have finally returned to the method by which I know it can certainly be done provided time enough be taken, which is by taking a porcelain tooth of the right color and grinding it down to fit,—a long, tedious piece of work. But if your assistant can do that for you, it will save considerable time, and I have had the best and most certain results by that method of any that I have tried. We all have to deal with atrophied spots on the teeth,—those tiny cavities and fissures extending across from mesial to distal surface,—and by taking the tiny pieces of porcelain that the White Company furnishes and one of the burs the circumference of which corresponds to the little disks of porcelain, you can grind down to fit accurately these tips of porcelain or porcelain rods which correspond exactly to the colors of the teeth. In making the larger porcelain fillings I burnish into the cavity a matrix of the thinnest platinum-foil that can be had; then, carefully withdrawing it, I turn Melotte's metal into that, and then taking a White or an Ash tooth, of the proper color, cement a piece of the porcelain of that tooth onto the metal and then grind,—and it is a grind to shape it to place, and then you can cement your inlay in place. I think many lay too much stress on How shall I cement that little piece of enamel to place? It doesn't require anything but a good cement to cement it to place with ease and accuracy.

Dr. Bert Russell, of Keene, N. H., showed at a dental meeting his method of putting in inlays,—and it was with the inlay rods, by the way.

These porcelain rods are cemented into a porte-polisher—sticky wax will answer; then, with the aid of the engine, grind to place and then cement to place and polish. It requires care and skill in dressing these little pieces of rod not to disturb the enamel, and it takes time to do it, but it can be done; it is done every day by him, and I adopt that same method without disturbing the enamel or injuring it in any way, and yet I can say that they do not break away from the cement very often. Once or twice I have had them dislodged by the aid of the tooth-brush, but it is very seldom that they give way. At the same time, I contend that all inlays, and not only inlays but gold fillings and crowns, should be examined from time to time with great care and painstaking to see that they have not failed. We know that frequently gold fillings fail, no matter how nicely put in, and we have also seen crowns give out because the best cement that we knew of had washed out. So we must not be surprised if inlays fail and have to be replaced.

I fear I have been disappointing to you in describing such a crude method, but certainly, with the Downie furnace and the bodies that have come to me, they have been quite as disappointing to me and, I have no doubt, to many of you. I have never yet found anything that will compare with the coloring as shown by the sample in the first firing, neither have I seen a body which would produce a color, I was going to say, twice alike.

*Dr. Eames.*—A patient of mine recently came from the hands of Dr. Jenkins, who had put in several inlays in teeth which I had not ordinarily considered suitable for such work. One of these was in the mesial surface of an upper lateral, the tooth being small with a thin cutting edge. The cavity, or rather the inlay, formed a part of the cutting edge, and I shall watch the durability of this piece of work with much interest. It was very nicely done and makes a satisfactory appearance in the mouth, but it can be seen five or six feet away.

The other inlay corresponded very nearly with the one described by Dr. Wilson, being in a lower bicuspid, labial surface, extending below the margin of the gum. It seems to me that gutta-percha or gold is preferable to inlays in such cases, or what I often prefer in such cavities extending far below the gum margin is to build up to the gum line with alloy, and finish with gold, cement, or gutta-percha at a subsequent sitting.

*Dr. Williams.*—I would like to ask Dr. Hamilton what cement he prefers?

*Dr. Hamilton.*—I do not profess to be any authority on cements for this purpose. I have tried several of them,—the Harvard, the Xenolite, and Weston's crown and bridge cement. The latter offers many points of advantage. It mixes very thin, indeed, and it is said to harden just as well as the slower-setting cements, but I do not think it has the strength. At present I prefer the Harvard.

*Dr. Daly.*—In using cement in these fissures and little pits of atrophy, to cement your porcelain to place you must not use your cement too stiff. Of course the porcelain is a non-conductor and is not affected by thermal changes, but I believe that all teeth should be protected by some varnish. The cavity should be coated with sandarac, or Canada balsam dissolved in chloroform, before you put your cement in. I feel that even amalgam fillings—if you use amalgam—can be made more comfortable to the tooth if the cavity is smeared with some kind of varnish before they are put in.

An advantage of the porcelain fillings besides their beauty, and I might sometimes say invisibility, is the fact that they are not affected by thermal changes.

*Dr. Gillett.*—For ten years or more I have made occasional use of porcelain fillings, using several different kinds. I have not been satisfied with any. Recently, I have been experimenting with the Jenkins outfit. I will pass round some samples showing some of the possibilities in color and form, and the consistency of the fused material. My estimate of the Jenkins outfit and process agrees very closely with that expressed by Dr. Briggs.

Dr. Jenkins has made feasible the gold-foil matrix and provided a low fusing material that is very much better than any that I have previously seen. It is far superior to the Richter, although fusing at nearly as low a temperature. The general color is good, and the shades do not change when fused with reasonable care. Material from the several bottles will practically always match the corresponding color sample. It resembles porcelain in appearance, but the thin edges look and act more like glass. It is hard enough for practical purposes, but I have found no difficulty in polishing it down with sand-paper disks. I think it sufficiently strong for labial and approximal cavities not exposed to very much stress, but should hesitate to use it on exposed corners. I may be mistaken in this, as Dr. Barrows, of Berlin, told me last summer that he was using it in crown- and bridge-work and found it satisfactory. Whether the profession abroad is as critical as to its standard of perfection in such work is a question for consideration. Dr. Hamilton's remark, "that it is surprising how much satisfaction a poor inlay will give," is very true. My first Jenkins's inlay was not satisfactory to me, but every one else is wonderfully happy over it. Others are entirely satisfactory to me, the color, fit, and finish being thoroughly good. I am satisfied that they will never be discovered so long as the cement maintains its integrity.

I would like to ask Dr. Daly how he would make an inlay from a porcelain rod to correspond to the larger inlay now going round.

I will call attention to the fact that for approximal work the wedging needs to be more (perhaps twice as much) as for gold work in order to get the matrix out and the inlay in. Often cavities will have to be opened more, one wall being opened back to the level of the floor of the cavity.

The fit depends entirely upon the accuracy of the gold matrix. If you make this accurately you get a perfect fit. It is very easy to spoil the matrix in tearing it out.

Dr. Joseph Head, at a recent clinic before the New York Odontological Society, showed a useful way of building out a corner of an incisor. He first crushed up a porcelain tooth of about the shade desired, and placed some of the crushed pieces in the matrix so they would stand out and give the desired contour. The low-fusing material was then added to fill the crevices and round out the surface.

Another point of interest was the fact that platinum-foil will be softer if annealed in charcoal, or, what is simpler for most of us, between sheets of asbestos, so keeping the air away while it is heated.

I have made crowns using the "Downie System,"—a porcelain facing attached to a gold cap and pin, and backed with Jenkins's body, the front of the band being also covered with the body. I

question its strength for such use, but think there are places in crown-work where it will be useful.

One important point to be mentioned is that this work, if well done, will take longer and cost more than to put in good gold fillings. My experience would indicate that it will often take twice the time. If you only prepare the matrix, it will be simpler but it will require an assistant with knowledge of the anatomy of the teeth, and good mechanical and artistic ability to warrant intrusting even the fusing to other hands.

*Dr. Daly.*—Referring to this case which Dr. Gillett asked me about, I could not do that with the porcelain rods. It is not possible. I have always resorted to the carver when such pieces as that came.

*Dr. Werner.*—I think Dr. Gillett brought out a point that has not been touched upon, and that is time. Will Dr. Hamilton please tell us his experience in that regard.

*Dr. Hamilton.*—At present it takes me twice as long to make a satisfactory inlay for a medium-sized cavity as it would for gold, but I think with more experience I can become a much more rapid worker.

*Dr. Wilson.*—I think the inlay rods are more or less unsatisfactory, and, as Dr. Gillett has just said, there are a great many irregular places where it is impossible to use them.

Something has just been said about "a perfect fit." The question is, What is a perfect fit? Gold fillings can be put in with such perfect joints that they cannot be detected with an explorer. On the other hand, it is a pretty difficult matter to put in a porcelain inlay where the joint cannot be detected with the aid of an explorer.

*Dr. Brackett.*—Before I begin my remarks on this subject, here is a specimen which helps to answer the doubts of the different gentlemen as to fit, and here is a magnifying glass under which it can be examined.

It seems to me that we are under very great obligations to Dr. Jenkins for what he has accomplished in providing us with a material that is fusible in a gold matrix,—a matrix which may be accurately adapted to the cavity,—and which seems to have great strength and excellent color.

We are under personal obligations to Dr. Charles Abbot, of Berlin, who has put this matter before us in such a comprehensive manner, and to Dr. E. D. Barrows, who has been Dr. Abbot's assistant for quite a number of years, and who brought to this country last summer the first apparatus of this kind which was seen here, and who was so good as to unpack the apparatus in Newport and give a demonstration of its working. I have a feeling of gratitude to these gentlemen for having done this much. I have also a personal feeling of gratitude to Dr. Barrows for procuring for me, as he did for Dr. Gillett, one of these outfits complete. I do not understand that there is any difficulty whatever in any of you gentlemen being similarly provided, except it be the disparity between the supply and demand. The apparatus itself retails in Germany at four hundred marks, very nearly one hundred dollars of our money, to which must be added the duty (which amounted, on the two that we received, to eighty-five dollars and fifty cents, or, say, forty-two dollars and seventy-five cents for one) and several other charges, so that by the time the apparatus is at your office it means an expense of one hundred and fifty to one hundred and fifty-five dollars. I have no doubt that any gentleman in this room who desires this apparatus, and will submit to Dr. Jenkins his application for one, can have it without any great delay.

In the demonstrations that Dr. Barrows made to us there was no laborious exertion required to do the work. There comes included in the apparatus an English blow-pipe, that is most easily operated by an oscillating motion, and I should say that the sum total of power needed in making an inlay is less than the sum total of power required in the making of a Richmond crown. The colors as shown are demonstrations from baked specimens. Dr. Barrows made the suggestion to me that additional colors could be made by the blending of the bodies as they come. The selecting of the body to be used is done by matching the tooth with the sample card; for instance, if the baked specimen on the card marked "No. 17" matches the place where the inlay is desired, the inlay that you make out of bottle No. 17 will match it equally well. As to the strength of the material, judging from the experiments which I have made, such as pounding it on the anvil with a hammer and crushing it, it seems to me to compare favorably with the strength of carved porcelain teeth such as I have got from Mr. Woodman.

It does not seem to me that there has been any overstating of this thing when accompanied by accuracy of manipulation and deliberation in the work. Dr. Barrows in his own practice, being a most energetic, indefatigable worker, follows this plan: He takes his impression, procuring his matrix and matching the color in regular office hours as the patient comes to the chair, and dismisses his patient for another sitting; then he makes his inlays quite largely with his own hands, and often in the evening, and is able in this way, with this long-continued labor himself, to do a great deal of work that could not well be all accomplished within ordinary office hours.

With reference to the objections that have been raised to the inaccurate fit of inlays that extend below the gum tissue, it seems to me that that is a mere trifling circumstance which could be readily overcome by building the tooth up to the gum level with some other and proper material and fitting the porcelain inlay to the rest of the cavity.

I hope that when this discussion is published it will be understood that we have an intelligent appreciation of the work of these dentists in Germany, and that we are grateful for what their labors have accomplished in our behalf.

*Dr. Daly.*—These corners and contours of Dr. Gillett's are certainly *restorations*, not inlays, but they come under inlay work. I am surprised to see the contour of it, and to see the density of that material. You know that the Downie body never furnished that. You can restore corners with the Allen body, or with the Consolidated bodies, but I should not expect to get such a contour. I have always felt that it was necessary to bake in pins until the past few years, but I now feel that the cement is tenacious enough to do all that is required of it.

*Dr. Gillett.*—Dr. Wilson has taken exception to my statement as to perfect fit, and perhaps it does need modification. What I had in mind was that the fit would be perfect from the stand-point of practical satisfaction,—that it would appear well. I have inlays in service which I cannot discover at a distance of three feet, although I know their exact location.

As regards utility, personally I would, for the present, limit it to those places in the mouth where we care more for appearance than for the very highest durability. So far as I know, it is the best we can do when the appearance of gold is objectionable. For such cavities, not exposed to heavy stress, I consider it the nearest to an ideal material that has yet been presented.

*Dr. Brackett.*—With reference to fit, Dr. Barrows showed that the restoration or inlay when it is pulled out of the matrix was all ready for setting without any grinding. It fits as if it were fused in the cavity itself. The matrix, being No. 30 or No. 40 gold-foil, provides for about as little cement as could possibly be used. A large restoration may have little grooves ground on the unexposed sides for the sake of giving a better hold to the cement.

*Dr. Hamilton.*—I have been rather interested for a year or two in the attachment of porcelain faces to platinum caps, and in making crowns from porcelain, using a platinum base. I have tried some with this Jenkins body, which seem strong even when the contour was built directly on the surface of the platinum.

In my investigations in that line, I have Mr. Woodman's authority for the statement that the low-fusing bodies will be harder to pull off or flake from platinum than the high-fusing bodies.

*Dr. Brackett.*—I would like Dr. Daly to tell us what cement he uses to try his porcelain rod in the cavity.

*Dr. Daly.*—I use the sticky wax of Fowler's, and also the shellac stick made by Claudius Ash. They say that it will attach the tooth to a rubber plate and it can be worn for a week.

*Dr. Wilson.*—In regard to Dr. Hamilton's allusion to baking a thin layer of porcelain over platinum, it is only very lately that I have seen one made in very much the same way. The thin porcelain was gradually becoming loose, and when the patient came to me it was about ready to drop off. Food had accumulated underneath the porcelain, giving it a black and unsightly appearance. This brings up rather an interesting question as to whether a low-fusing body will adhere more closely to platinum than a high-fusing body. In making the tooth over, a high-fusing body was used. I would like to hear Dr. Williams's views on this point.

*Dr. Williams.*—I should say it would be apt to adhere to the platinum all right, but I should not expect it to be as strong as the high-fusing body.

Harry E. Cutter, D.D.S.,
*Editor American Academy of Dental Science.*

